# Supplementary material for: Excess mortality of infected ectotherms induced by warming depends on pathogen kingdom and evolutionary history
Source: PLoS Biol. 2024 Nov 18;22(11):e3002900. doi: 10.1371/journal.pbio.3002900 (PMC11611255; doi:10.1371/journal.pbio.3002900)
Supplement: S1 Text — List of studies included PRISMA-EcoEvo Checklist. (DOCX) [file pbio.3002900.s001.docx]

**Supporting Information for**

Excess mortality of infected ectotherms induced by warming depends on pathogen kingdom and evolutionary history

J. Li^1,4^, N. Guttmann^2, 3^, G.C. Drew^1,6^, T.E. Hector^1^, J. Wolinska^2, 3^, K.C. King^1,4,5^

1. Department of Biology, University of Oxford, Oxford OX1 3SZ, United Kingdom
2. Department of Evolutionary and Integrative Ecology, Leibniz Institute of Freshwater Ecology and Inland Fisheries (IGB), Berlin, Germany
3. Department of Biology, Chemistry, Pharmacy, Institute of Biology, Freie Universität Berlin (FU), Berlin, Germany
4. Department of Zoology, University of British Columbia, Vancouver, Canada
5. Department of Microbiology & Immunology, University of British Columbia, Vancouver, Canada
6. Collegium Helveticum, The joint Institute for Advanced Studies (IAS) of the ETH Zurich, The University of Zurich, &The Zurich University of the Arts, Zurich, Switzerland

**Email:**  [jingdi.li@biology.ox.ac.uk](mailto:jingdi.li@biology.ox.ac.uk)

**This file (S1 Text) includes:**

List of studies included

PRISMA-EcoEvo Checklist

**List of papers included**

1. Aatif, H.M., Hanif, M.S., Raheel, M. et al. Temperature dependent virulence of the entomopathogenic nematodes against immatures of the oriental fruit fly, Bactrocera dorsalis Hendel (Diptera: Tephritidae). Egypt J Biol Pest Control 30, 42 (2020). https://doi.org/10.1186/s41938-020-00248-7

2. BALLY, M. and GARRABOU, J. (2007), Thermodependent bacterial pathogens and mass mortalities in temperate benthic communities: a new case of emerging disease linked to climate change. Global Change Biology, 13: 2078-2088. https://doi.org/10.1111/j.1365-2486.2007.01423.x

3. Brand, M. D., Hill, R. D., Brenes, R., Chaney, J. C., Wilkes, R. P., Grayfer, L., Miller, D. L., & Gray, M. J. (2016). Water Temperature Affects Susceptibility to Ranavirus. EcoHealth, 13(2), 350–359. https://doi.org/10.1007/s10393-016-1120-1

4. Bugeme, D. M., Knapp, M., Boga, H. I., Wanjoya, A. K., & Maniania, N. K. (2009). Influence of temperature on virulence of fungal isolates of Metarhizium anisopliae and Beauveria bassiana to the two-spotted spider mite Tetranychus urticae. Mycopathologia, 167(4), 221–227. https://doi.org/10.1007/s11046-008-9164-6

5. Bugeme, D. M., Maniania, N. K., Knapp, M., & Boga, H. I. (2008). Effect of temperature on virulence of Beauveria bassiana and Metarhizium anisopliae isolates to Tetranychus evansi. Experimental & applied acarology, 46(1-4), 275–285. https://doi.org/10.1007/s10493-008-9179-1

6. Buisson, C., Gohar, M., Huillet, E., & Nielsen-LeRoux, C. (2019). Bacillus thuringiensis Spores and Vegetative Bacteria: Infection Capacity and Role of the Virulence Regulon PlcR Following Intrahaemocoel Injection of Galleria mellonella. Insects, 10(5), 129. https://doi.org/10.3390/insects10050129

7. Rao, C. Uma Maheswara, K. Uma Devi kumadevi@eth.net & P. Akbar Ali Khan (2006) Effect of combination treatment with entomopathogenic fungi Beauveria bassiana and Nomuraea rileyi (Hypocreales) on Spodoptera litura (Lepidoptera: Noctuidaeae), Biocontrol Science and Technology, 16:3, 221-232, DOI: 10.1080/09583150500335632

8. Chideroli, R.T., Amoroso, N., Mainardi, R.M., Suphoronski, S.A., de Padua, S.B., Alfieri, A.F., Alfieri, A.A., Mosela, M. et al. (2017) Emergence of a new multidrug-resistant and highly virulent serotype of Streptococcus agalactiae in fish farms from Brazil. Aquaculture 479, 45-51. 10.1016/j.aquaculture.2017.05.013

9. Tesfaye D. and E. Seyoum "Studies on the Pathogenicity of Native Entomopathogenic Fungal Isolates on the Cotton/Melon Aphid, Aphis gossypii (Homoptera: Aphididae) Glover under Different Temperature Regimes," African Entomology 18(2), 302-312, (1 September 2010). https://doi.org/10.4001/003.018.0215

10. Tesfaye Dawit Degefu, Emiru Seyoum Yeshanew & Geremew Terefe Gashawbeza (2014) Efficacy of dose-dependent indigenous microbial insecticides against cotton aphid, Aphis gossypii Glover (Homoptera: Aphididae) at various temperature regimes, International Journal of Pest Management, 60:3, 173-179, DOI: 10.1080/09670874.2014.951101

11. De Silva, P. M., Chong, P., Fernando, D. M., Westmacott, G., & Kumar, A. (2017). Effect of Incubation Temperature on Antibiotic Resistance and Virulence Factors of Acinetobacter baumannii ATCC 17978. Antimicrobial agents and chemotherapy, 62(1), e01514-17. https://doi.org/10.1128/AAC.01514-17

12. Delisle, L., Petton, B., Burguin, J. F., Morga, B., Corporeau, C., & Pernet, F. (2018). Temperature modulate disease susceptibility of the Pacific oyster Crassostrea gigas and virulence of the Ostreid herpesvirus type 1. Fish & shellfish immunology, 80, 71–79. https://doi.org/10.1016/j.fsi.2018.05.056

13. Yuksel E. et al. Potential of four Turkish isolates of entomopathogenic nematodes against three major stored products insect pests. J. Stored Prod. Res. (2019). https://doi.org/10.1016/j.jspr.2019.08.003

14. Ekesi, S., Maniania, N.K., & Ampong‐Nyarko, K. (1999). Effect of Temperature on Germination, Radial Growth and Virulence of Metarhizium anisopliae and Beauveria bassiana on Megalurothrips sjostedti. Biocontrol Science and Technology, 9, 177-185.

15. Del Valle Eleodoro E., Emilia I. Balbi, Paola Lax, Juan Rondán Dueñas & Marcelo E. Doucet (2014) Ecological aspects of an isolate of Steinernema diaprepesi (Rhabditida: Steinernematidae) from Argentina, Biocontrol Science and Technology, 24:6, 690-704, DOI: 10.1080/09583157.2014.890171

16. Ferguson, L. V., & Sinclair, B. J. (2020). Thermal Variability and Plasticity Drive the Outcome of a Host-Pathogen Interaction. The American naturalist, 195(4), 603–615. https://doi.org/10.1086/707545

17. Garcia-Solache, M. A., Izquierdo-Garcia, D., Smith, C., Bergman, A., & Casadevall, A. (2013). Fungal virulence in a lepidopteran model is an emergent property with deterministic features. mBio, 4(3), e00100–e113. https://doi.org/10.1128/mBio.00100-13

18. Garcia, A. R., Rocha, A. P., Moreira, C. C., Rocha, S. L., Guarneri, A. A., & Elliot, S. L. (2016). Screening of Fungi for Biological Control of a Triatomine Vector of Chagas Disease: Temperature and Trypanosome Infection as Factors. PLoS neglected tropical diseases, 10(11), e0005128. https://doi.org/10.1371/journal.pntd.0005128

19. Grewal, P. S., Gaugler, R., & Shupe, C. (1996). Rapid Changes in Thermal Sensitivity of Entomopathogenic Nematodes in Response to Selection at Temperature Extremes. Journal of invertebrate pathology, 68(1), 65–73. https://doi.org/10.1006/jipa.1996.0059

20. Heinig, R. L., Paaijmans, K. P., Hancock, P. A., & Thomas, M. B. (2015). The potential for fungal biopesticides to reduce malaria transmission under diverse environmental conditions. The Journal of applied ecology, 52(6), 1558–1566. https://doi.org/10.1111/1365-2664.12522

21. Hu WJ, Hou RFN, Talekar NS. Pathogenicity of Beauveria bassiana to Riptortus linearis (Hemiptera: Coreidae), a pest of soybean. Appl Entomol Zool. 1996; 31: 187–194. 10.1303/aez.31.187

22. Huang, X., Li, D., Xi, L., & Mylonakis, E. (2015). Galleria mellonella Larvae as an Infection Model for Penicillium marneffei. Mycopathologia, 180(3-4), 159–164. https://doi.org/10.1007/s11046-015-9897-y

23. Jiang, M., Chen, Z. G., Zheng, J., & Peng, B. (2019). Metabolites-Enabled Survival of Crucian Carps Infected by Edwardsiella tarda in High Water Temperature. Frontiers in immunology, 10, 1991. https://doi.org/10.3389/fimmu.2019.01991

24. Jiang, Q., Shi, L., Ke, C., You, W., & Zhao, J. (2013). Identification and characterization of Vibrio harveyi associated with diseased abalone Haliotis diversicolor. Diseases of aquatic organisms, 103(2), 133–139. https://doi.org/10.3354/dao02572

25. Keerio, A.U., Nazir, T., Abdulle, Y.A. et al. In vitro pathogenicity of the fungi Beauveria bassiana and Lecanicillium lecanii at different temperatures against the whitefly, Bemisia tabaci (Genn.) (Hemiptera: Aleyrodidae). Egypt J Biol Pest Control 30, 41 (2020). https://doi.org/10.1186/s41938-020-00247-8

26. Kerchev, I.A., Kryukov, V.Y., Yaroslavtseva, O.N. et al. The first data on fungal pathogens (ascomycota, hypocreales) in the invasive populations of four-eyed fir bark beetle Polygraphus proximus Blandf.. Russ J Biol Invasions 8, 34–40 (2017). https://doi.org/10.1134/S2075111717010040

27. Kikankie, C.K., Brooke, B.D., Knols, B.G. et al. The infectivity of the entomopathogenic fungus Beauveria bassiana to insecticide-resistant and susceptible Anopheles arabiensis mosquitoes at two different temperatures. Malar J 9, 71 (2010). https://doi.org/10.1186/1475-2875-9-71

28. Kirk, D., Jones, N., Peacock, S., Phillips, J., Molnár, P. K., Krkošek, M., & Luijckx, P. (2018). Empirical evidence that metabolic theory describes the temperature dependency of within-host parasite dynamics. PLoS biology, 16(2), e2004608. https://doi.org/10.1371/journal.pbio.2004608

29. Kryukov VY, Tomilova OG, Yaroslavtseva ON, Wen T-C, Kryukova NA, Polenogova OV, Tokarev YS, Glupov VV (2018) Temperature adaptations of Cordyceps militaris, impact of host thermal biology and immunity on mycosis development. Fungal Ecol 35:98–107. https://doi.org/10.1016/j.funeco.2018.07.003

30. Larsen, A. K., Nymo, I. H., Sørensen, K. K., Seppola, M., Rødven, R., Jiménez de Bagüés, M. P., Al Dahouk, S., & Godfroid, J. (2018). Concomitant Temperature Stress and Immune Activation may Increase Mortality Despite Efficient Clearance of an Intracellular Bacterial Infection in Atlantic Cod. Frontiers in microbiology, 9, 2963. https://doi.org/10.3389/fmicb.2018.02963

31. Lokmer, A., & Mathias Wegner, K. (2015). Hemolymph microbiome of Pacific oysters in response to temperature, temperature stress and infection. The ISME journal, 9(3), 670–682. https://doi.org/10.1038/ismej.2014.160

32. Long et al. 2000. Influence of temperature on the infectivity of entomopathogenic nematodes (Steinernema and Heterorhabditis spp.) to larvae and pupae of the vine weevil Otiorhynchus sulcatus (Coleoptera: Curculionidae). Nematology. Volumn 2: Issue 3, pages 309-317, https://doi.org/10.1163/156854100509187

33. López, J. R., Lorenzo, L., Marcelino-Pozuelo, C., Marin-Arjona, M. C., & Navas, J. I. (2017). Pseudomonas baetica: pathogenicity for marine fish and development of protocols for rapid diagnosis. FEMS microbiology letters, 364(3), 10.1093/femsle/fnw286. https://doi.org/10.1093/femsle/fnw286

34. Mesa-Arango, A. C., Forastiero, A., Bernal-Martínez, L., Cuenca-Estrella, M., Mellado, E., & Zaragoza, O. (2013). The non-mammalian host Galleria mellonella can be used to study the virulence of the fungal pathogen Candida tropicalis and the efficacy of antifungal drugs during infection by this pathogenic yeast. Medical mycology, 51(5), 461–472. https://doi.org/10.3109/13693786.2012.737031

35. Phuoc N.N. et al. Effect of strain and enviromental conditions on the virulence of Streptococcus agalactiae (Group B Streptococcus; GBS) in red tilapia (Oreochromis sp.) Aqua (2021). https://doi.org/10.1016/j.aquaculture.2020.736256

36. Öğretmen, A.; Yüksel, E.; Canhilal, R. Susceptibility of larvae of wireworms (Agriotes spp.) (Coleoptera: Elateridae) to some Turkish isolates of entomopathogenic nematodes under laboratory and field conditions. Biol. Control 2020, 149, 104320.

37. Páez, D. J., Powers, R. L., Jia, P., Ballesteros, N., Kurath, G., Naish, K. A., & Purcell, M. K. (2021). Temperature Variation and Host Immunity Regulate Viral Persistence in a Salmonid Host. Pathogens (Basel, Switzerland), 10(7), 855. https://doi.org/10.3390/pathogens10070855

38. Petersen, L. M., & Tisa, L. S. (2012). Influence of temperature on the physiology and virulence of the insect pathogen Serratia sp. Strain SCBI. Applied and environmental microbiology, 78(24), 8840–8844. https://doi.org/10.1128/AEM.02580-12

39. Réjasse, A., Gilois, N., Barbosa, I., Huillet, E., Bevilacqua, C., Tran, S., Ramarao, N., Stenfors Arnesen, L. P., & Sanchis, V. (2012). Temperature-dependent production of various PlcR-controlled virulence factors in Bacillus weihenstephanensis strain KBAB4. Applied and environmental microbiology, 78(8), 2553–2561. https://doi.org/10.1128/AEM.07446-11

40. Rodrigues, J.deO., Lorenzo, M. G., Martins-Filho, O. A., Elliot, S. L., & Guarneri, A. A. (2016). Temperature and parasite life-history are important modulators of the outcome of Trypanosoma rangeli-Rhodnius prolixus interactions. Parasitology, 143(11), 1459–1468. https://doi.org/10.1017/S0031182016001062

41. Saif-ur-Rehman, Zheng, J., Ahmed, N., Feng, J., & Wang, D. (2019). POTENTIAL OF FOUR ENTOMOPATHOGENIC FUNGI ISOLATES AS BIOLOGICAL CONTROL AGENTS AGAINST TWO APHID SPECIES UNDER LABORATORY CONDITIONS. 10.21162/PAKJAS/19.8582

42. Schade, F. M., Shama, L. N., & Wegner, K. M. (2014). Impact of thermal stress on evolutionary trajectories of pathogen resistance in three-spined stickleback (Gasterosteus aculeatus). BMC evolutionary biology, 14, 164. https://doi.org/10.1186/s12862-014-0164-5

43. Seman, B. G., Moore, J. L., Scherer, A. K., Blair, B. A., Manandhar, S., Jones, J. M., & Wheeler, R. T. (2018). Yeast and Filaments Have Specialized, Independent Activities in a Zebrafish Model of Candida albicans Infection. Infection and immunity, 86(10), e00415-18. https://doi.org/10.1128/IAI.00415-18

44. Sepahi A., Marzieh Heidarieh, Alireza Mirvaghefi, Gholam Reza Rafiee, Mehrnoush Farid & Najmeh Sheikhzadeh. Effects of Water Temperature on the Susceptibility of Rainbow Trout to Streptococcus agalactiae. Acta Scientiae Veterinariae, 2013. 41: 1097

45. Shapiro-Ilan, D. I., Blackburn, D., Duncan, L., El-Borai, F. E., Koppenhöfer, H., Tailliez, P., & Adams, B. J. (2014). Characterization of Biocontrol Traits in Heterorhabditis floridensis: A Species with Broad Temperature Tolerance. Journal of nematology, 46(4), 336–345.

46. Shaurub, E. H., Soliman, N. A., Hashem, A. G., & Abdel-Rahman, A. M. (2015). Infectivity of Four Entomopathogenic Nematodes in Relation to Environmental Factors and Their Effects on the Biochemistry of the Medfly Ceratitis capitata (Wied.) (Diptera: Tephritidae). Neotropical entomology, 44(6), 610–618. https://doi.org/10.1007/s13744-015-0332-3

47. Suchman, E. L., Kononko, A., Plake, E., Doehling, M., Kleker, B., Black, W. C., et al. (2006). Effects of AeDNV infection on Aedes aegypti lifespan and reproduction. Biol. Control 39, 465–473. doi: 10.1016/j.biocontrol.2006.05.001

48. Svedese, V. M., Lima, E. Á. de L. A., & Porto, A. L. F.. (2013). Horizontal transmission and effect of the temperature in pathogenicity of Beauveria bassiana against Diatraea saccharalis (Lepidoptera: Crambidae). Brazilian Archives of Biology and Technology, 56(3), 413–419. https://doi.org/10.1590/S1516-89132013000300009

49. Trdan, S., Vidrih, M., Andjus, L. et al. Activity of four entomopathogenic nematode species against different developmental stages of Colorado potato beetle, Leptinotarsa decemlineata (Coleoptera, Chrysomelidae). Helminthologia 46, 14–20 (2009). https://doi.org/10.2478/s11687-009-0003-1

50. Ushijima, B., Richards, G. P., Watson, M. A., Schubiger, C. B., & Häse, C. C. (2018). Factors affecting infection of corals and larval oysters by Vibrio coralliilyticus. PloS one, 13(6), e0199475. https://doi.org/10.1371/journal.pone.0199475

51. Ushijima, B., Videau, P., Burger, A. H., Shore-Maggio, A., Runyon, C. M., Sudek, M., Aeby, G. S., & Callahan, S. M. (2014). Vibrio coralliilyticus strain OCN008 is an etiological agent of acute Montipora white syndrome. Applied and environmental microbiology, 80(7), 2102–2109. https://doi.org/10.1128/AEM.03463-13

52. Vale, P. F., & Little, T. J. (2009). Measuring parasite fitness under genetic and thermal variation. Heredity, 103(2), 102–109. https://doi.org/10.1038/hdy.2009.54

53. Vale, P. F., Stjernman, M., & Little, T. J. (2008). Temperature-dependent costs of parasitism and maintenance of polymorphism under genotype-by-environment interactions. Journal of evolutionary biology, 21(5), 1418–1427. https://doi.org/10.1111/j.1420-9101.2008.01555.x

54. Vater, A., Byrne, B. A., Marshman, B. C., Ashlock, L. W., & Moore, J. D. (2018). Differing responses of red abalone (Haliotis rufescens) and white abalone (H. sorenseni) to infection with phage-associated Candidatus Xenohaliotis californiensis. PeerJ, 6, e5104. https://doi.org/10.7717/peerj.5104

55. Vezzulli, L., Previati, M., Pruzzo, C., Marchese, A., Bourne, D. G., Cerrano, C., & VibrioSea Consortium (2010). Vibrio infections triggering mass mortality events in a warming Mediterranean Sea. Environmental microbiology, 12(7), 2007–2019. https://doi.org/10.1111/j.1462-2920.2010.02209.x

56. Waki T, Yoshinaga T. Suppressive effects of low salinity and low temperature on in-vivo propagation of the protozoan Perkinsus olseni in Manila clam. Fish Pathol. (2015) 50:16–22. 10.3147/jsfp.50.16

57. Wekesa, V. W., Moraes, G. J., Ortega, E. M., & Delalibera, I., Jr (2010). Effect of temperature on sporulation of Neozygites floridana isolates from different climates and their virulence against the tomato red spider mite, Tetranychus evansi. Journal of invertebrate pathology, 103(1), 36–42. https://doi.org/10.1016/j.jip.2009.10.003

58. Yang B., S. Zhai, X. Li, J. Tian, Q. Li, H. Shan, S. Liu. Identification of Vibrio alginolyticus as a causative pathogen associated with mass summer mortality of the Pacific Oyster (Crassostrea gigas) in China. Aquaculture, 535 (2021), Article 736363, 10.1016/j.aquaculture.2021.736363

59. Yeo, H., Pell, J. K., Alderson, P. G., Clark, S. J., & Pye, B. J. (2003). Laboratory evaluation of temperature effects on the germination and growth of entomopathogenic fungi and on their pathogenicity to two aphid species. Pest management science, 59(2), 156–165. https://doi.org/10.1002/ps.622

60. Wei Z. et al. Isolation and characterization of Vibrio harveyi as a major pathogen associated with mass mortalities of ark clam, Scapharca broughtonii, in summer. Aquaculture. (2019) 10.1016/j.aquaculture.2019.734248

| **PRISMA-EcoEvo Checklist**  Following O'Dea, R.E., Lagisz, M., Jennions, M.D., Koricheva, J., Noble, D.W., Parker, T.H., Gurevitch, J., Page, M.J., Stewart, G., Moher, D. and Nakagawa, S. (2021), Preferred reporting items for systematic reviews and meta-analyses in ecology and evolutionary biology: a PRISMA extension. Biol Rev. doi:10.1111/brv.12721 | | | | |
| --- | --- | --- | --- | --- |
| **Checklist item** | **Sub-item number** | **Sub-item** | **Reported by authors?** | **Notes** |
| Title and abstract | 1.1 | Identify the review as a systematic review, meta-analysis, or both | Yes |  |
|  | 1.2 | Summarise the aims and scope of the review | Yes |  |
|  | 1.3 | Describe the data set | Yes |  |
|  | 1.4 | State the results of the primary outcome | Yes |  |
|  | 1.5 | State conclusions | Yes |  |
|  | 1.6 | State limitations | Yes |  |
| Aims and questions | 2.1 | Provide a rationale for the review | Yes |  |
|  | 2.2 | Reference any previous reviews or meta-analyses on the topic | Yes |  |
|  | 2.3 | State the aims and scope of the review (including its generality) | Yes |  |
|  | 2.4 | State the primary questions the review addresses (e.g. which moderators were tested) | Yes |  |
|  | 2.5 | Describe whether effect sizes were derived from experimental and/or observational comparisons | Yes |  |
| Review registration | 3.1 | Register review aims, hypotheses (if applicable), and methods in a time-stamped and publicly accessible archive and provide a link to the registration in the methods section of the manuscript. Ideally registration occurs before the search, but it can be done at any stage before data analysis. | No |  |
|  | 3.2 | Describe deviations from the registered aims and methods | No |  |
|  | 3.3 | Justify deviations from the registered aims and methods | No |  |
| Eligibility criteria | 4.1 | Report the specific criteria used for including or excluding studies when screening titles and/or abstracts, and full texts, according to the aims of the systematic review (e.g. study design, taxa, data availability) | Yes |  |
|  | 4.2 | Justify criteria, if necessary (i.e. not obvious from aims and scope) | Yes |  |
| Finding studies | 5.1 | Define the type of search (e.g. comprehensive search, representative sample) | Yes |  |
|  | 5.2 | State what sources of information were sought (e.g. published and unpublished studies, personal communications) | Yes |  |
|  | 5.3 | Include, for each database searched, the exact search strings used, with keyword combinations and Boolean operators | Yes |  |
|  | 5.4 | Provide enough information to repeat the equivalent search (if possible), including the timespan covered (start and end dates) | Yes |  |
| Study selection | 6.1 | Describe how studies were selected for inclusion at each stage of the screening process (e.g. use of decision trees, screening software) | Yes | Manually |
|  | 6.2 | Report the number of people involved and how they contributed (e.g. independent parallel screening) | No |  |
| Data collection process | 7.1 | Describe where in the reports data were collected from (e.g. text or figures) | Yes |  |
|  | 7.2 | Describe how data were collected (e.g. software used to digitize figures, external data sources) | Yes |  |
|  | 7.3 | Describe moderator variables that were constructed from collected data (e.g. number of generations calculated from years and average generation time) | Yes |  |
|  | 7.4 | Report how missing or ambiguous information was dealt with during data collection (e.g. authors of original studies were contacted for missing descriptive statistics, and/or effect sizes were calculated from test statistics) | Yes | Contacted authors when data was missing |
|  | 7.5 | Report who collected data | Yes |  |
|  | 7.6 | State the number of extractions that were checked for accuracy by co-authors | No | Co-authors randomly checked records for accuracy |
| Data items | 8.1 | Describe the key data sought from each study | Yes |  |
|  | 8.2 | Describe items that do not appear in the main results, or which could not be extracted due to insufficient information | Yes |  |
|  | 8.3 | Describe main assumptions or simplifications that were made (e.g. categorising both ‘length’ and ‘mass’ as ‘morphology’) | Yes |  |
|  | 8.4 | Describe the type of replication unit (e.g. individuals, broods, study sites) | Yes |  |
| Assessment of individual study quality | 9.1 | Describe whether the quality of studies included in the systematic review or meta-analysis was assessed (e.g. blinded data collection, reporting quality, experimental *versus* observational) | No | All included studies were experimental |
|  | 9.2 | Describe how information about study quality was incorporated into analyses (e.g. meta-regression and/or sensitivity analysis) | No |  |
| Effect size measures | 10.1 | Describe effect size(s) used | Yes |  |
|  | 10.2 | Provide a reference to the equation of each calculated effect size (e.g. standardised mean difference, log response ratio) and (if applicable) its sampling variance | Yes |  |
|  | 10.3 | If no reference exists, derive the equations for each effect size and state the assumed sampling distribution(s) | NA |  |
| Missing data | 11.1 | Describe any steps taken to deal with missing data during analysis (e.g. imputation, complete case, subset analysis) | No | Data that could not be obtained from paper or from authors was excluded |
|  | 11.2 | Justify the decisions made to deal with missing data | NA |  |
| Meta-analytic model description | 12.1 | Describe the models used for synthesis of effect sizes | Yes |  |
|  | 12.2 | The most common approach in ecology and evolution will be a random-effects model, often with a hierarchical/multilevel structure. If other types of models are chosen (e.g. common/fixed effects model, unweighted model), provide justification for this choice | Random-effects models were used |  |
| Software | 13.1 | Describe the statistical platform used for inference (e.g. *R*) | Yes |  |
|  | 13.2 | Describe the packages used to run models | Yes |  |
|  | 13.3 | Describe the functions used to run models | Yes |  |
|  | 13.4 | Describe any arguments that differed from the default settings | Yes |  |
|  | 13.5 | Describe the version numbers of all software used | Yes |  |
| Non-independence | 14.1 | Describe the types of non-independence encountered (e.g. phylogenetic, spatial, multiple measurements over time) | Yes |  |
|  | 14.2 | Describe how non-independence has been handled | Yes |  |
|  | 14.3 | Justify decisions made | Yes | We justified that why we didn’t include phylogenetic tree as a random factor |
| Meta-regression and model selection | 15.1 | Provide a rationale for the inclusion of moderators (covariates) that were evaluated in meta-regression models | Yes |  |
|  | 15.2 | Justify the number of parameters estimated in models, in relation to the number of effect sizes and studies (e.g. interaction terms were not included due to insufficient sample sizes) | Yes |  |
|  | 15.3 | Describe any process of model selection | Yes |  |
| Publication bias and sensitivity analyses | 16.1 | Describe assessments of the risk of bias due to missing results (e.g. publication, time-lag, and taxonomic biases) | Yes |  |
|  | 16.2 | Describe any steps taken to investigate the effects of such biases (if present) | Yes |  |
|  | 16.3 | Describe any other analyses of robustness of the results, e.g. due to effect size choice, weighting or analytical model assumptions, inclusion or exclusion of subsets of the data, or the inclusion of alternative moderator variables in meta-regressions | Yes |  |
| Clarification of *post hoc* analyses | 17.1 | When hypotheses were formulated after data analysis, this should be acknowledged. | We did not formulate hypotheses after data analysis |  |
| Metadata, data, and code | 18.1 | Share metadata (i.e. data descriptions) | Yes |  |
|  | 18.2 | Share data required to reproduce the results presented in the manuscript | Yes |  |
|  | 18.3 | Share additional data, including information that was not presented in the manuscript (e.g. raw data used to calculate effect sizes, descriptions of where data were located in papers) | Yes |  |
|  | 18.4 | Share analysis scripts (or, if a software package with graphical user interface (GUI) was used, then describe full model specification and fully specify choices) | Yes |  |
| Results of study selection process | 19.1 | Report the number of studies screened | Yes |  |
|  | 19.2 | Report the number of studies excluded at each stage of screening | Yes |  |
|  | 19.3 | Report brief reasons for exclusion from the full text stage | Yes |  |
|  | 19.4 | Present a Preferred Reporting Items for Systematic Reviews and Meta-Analyses (PRISMA)-like flowchart (www.prisma-statement.org). | Yes |  |
| Sample sizes and study characteristics | 20.1 | Report the number of studies and effect sizes for data included in meta-analyses | Yes |  |
|  | 20.2 | Report the number of studies and effect sizes for subsets of data included in meta-regressions | Yes |  |
|  | 20.3 | Provide a summary of key characteristics for reported outcomes (either in text or figures; e.g. one quarter of effect sizes reported for vertebrates and the rest invertebrates) | Yes |  |
|  | 20.4 | Provide a summary of limitations of included moderators (e.g. collinearity and overlap between moderators) | Yes |  |
|  | 20.5 | Provide a summary of characteristics related to individual study quality (risk of bias) | Yes | We assessed sample size for individual study |
| Meta-analysis | 21.1 | Provide a quantitative synthesis of results across studies, including estimates for the mean effect size, with confidence/credible intervals | Yes |  |
| Heterogeneity | 22.1 | Report indicators of heterogeneity in the estimated effect (e.g. *I*^2^, *tau*^2^ and other variance components) | No |  |
| Meta-regression | 23.1 | Provide estimates of meta-regression slopes (i.e. regression coefficients) and confidence/credible intervals | Yes |  |
|  | 23.2 | Include estimates and confidence/credible intervals for all moderator variables that were assessed (i.e. complete reporting) | Yes |  |
|  | 23.3 | Report interactions, if they were included | Yes |  |
|  | 23.4 | Describe outcomes from model selection, if done (e.g. R2 and AIC) | No |  |
| Outcomes of publication bias and sensitivity analyses | 24.1 | Provide results for the assessments of the risks of bias (e.g. Egger's regression, funnel plots) | Yes | We used sample size to assess publication bias. |
|  | 24.2 | Provide results for the robustness of the review's results (e.g. subgroup analyses, meta-regression of study quality, results from alternative methods of analysis, and temporal trends) | Yes |  |
| Discussion | 25.1 | Summarise the main findings in terms of the magnitude of effect | Yes |  |
|  | 25.2 | Summarise the main findings in terms of the precision of effects (e.g. size of confidence intervals, statistical significance) | Yes |  |
|  | 25.3 | Summarise the main findings in terms of their heterogeneity | Yes |  |
|  | 25.4 | Summarise the main findings in terms of their biological/practical relevance | Yes |  |
|  | 25.5 | Compare results with previous reviews on the topic, if available | Yes | We haven’t found a systematic review on this topic |
|  | 25.6 | Consider limitations and their influence on the generality of conclusions, such as gaps in the available evidence (e.g. taxonomic and geographical research biases) | Yes |  |
| Contributions and funding | 26.1 | Provide names, affiliations, and funding sources of all co-authors | Yes |  |
|  | 26.2 | List the contributions of each co-author | Yes |  |
|  | 26.3 | Provide contact details for the corresponding author | Yes |  |
|  | 26.4 | Disclose any conflicts of interest | Yes |  |
| References | 27.1 | Provide a reference list of all studies included in the systematic review or meta-analysis | Yes |  |
|  | 27.2 | List included studies as referenced sources (e.g. rather than listing them in a table or supplement) | No | All included studies are referenced in the supplement |
