# Supplementary figures and images for: Excess mortality of infected ectotherms induced by warming depends on pathogen kingdom and evolutionary history
Source: PLoS Biol. 2024 Nov 18;22(11):e3002900. doi: 10.1371/journal.pbio.3002900 (PMC11611255; doi:10.1371/journal.pbio.3002900)

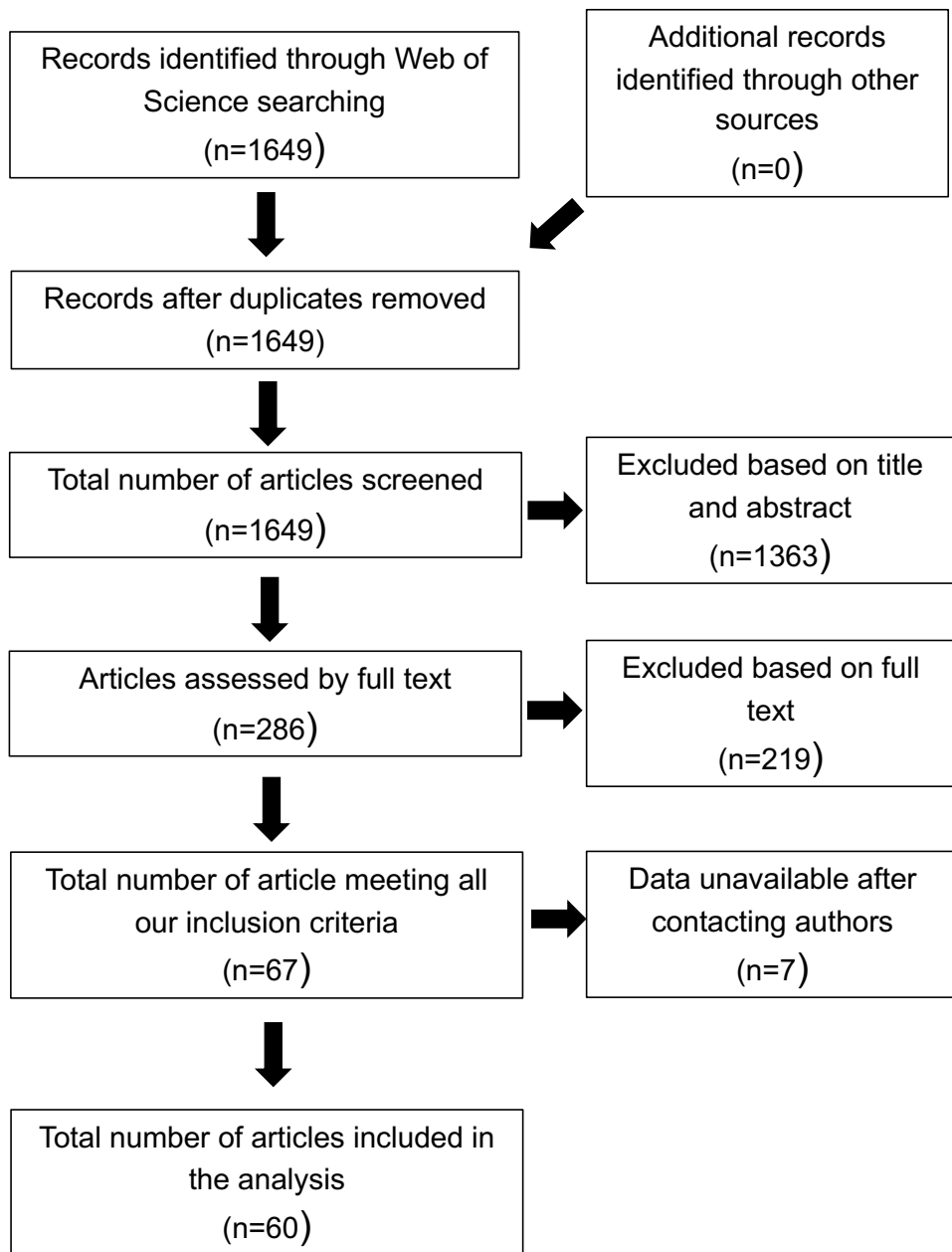

Supplement: S1 Fig — The 1,649 records from a Web of Science search were screened based on our criteria, resulting in the 60 total articles included in the meta-analysis; n represents the number of articles. (PDF) [file pbio.3002900.s002.pdf]

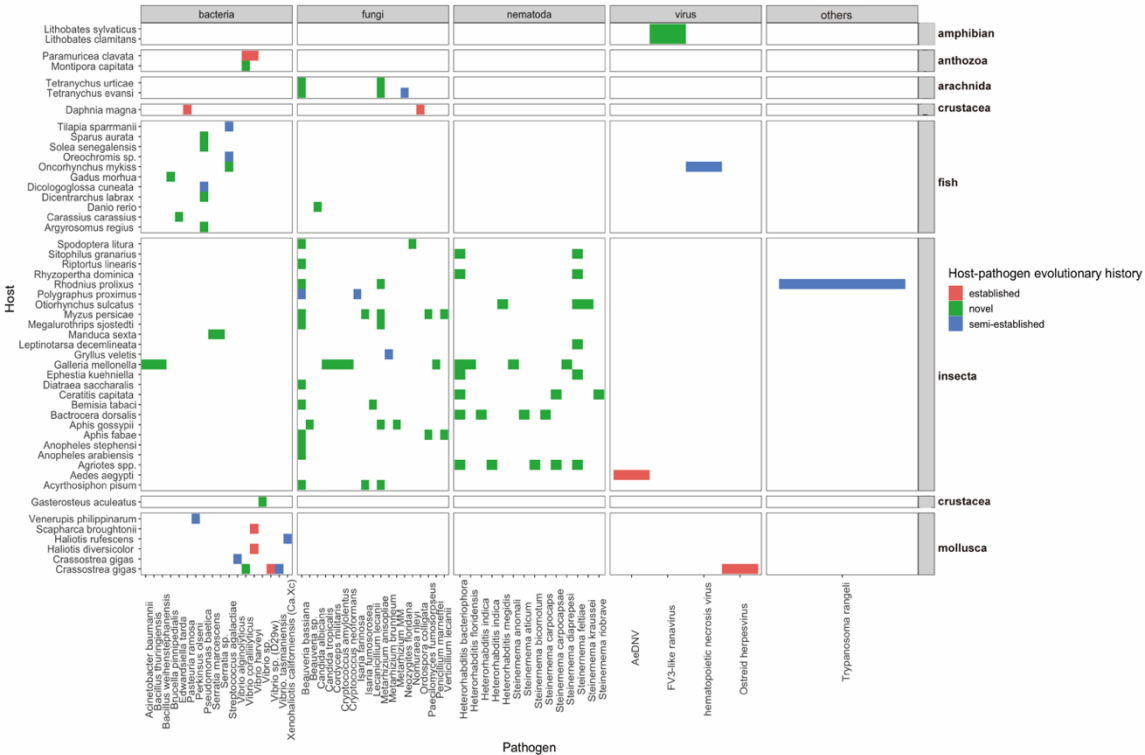

Supplement: S2 Fig — Each is colored by the evolutionary history of the host–pathogen system (established, semi-established, and novel as defined in the main text). The data and code needed to generate this figure can be found in https://doi.org/10.6084/m9.figshare.22060646.v7. (PDF) [file pbio.3002900.s003.pdf]

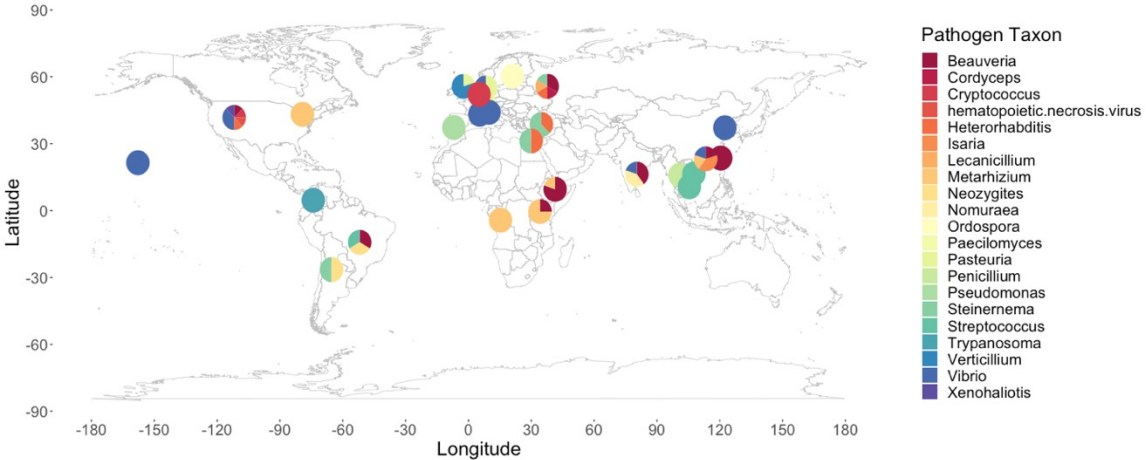

Supplement: S3 Fig — Some pathogens were collected years ago and may have adapted to lab environment. Different colors represent pathogen taxa (shown on genus-level when available). Collection sites data were variable in their geographic specificity, ranging from precise coordinates to broader regions such as cities and countries, depending on details reported in the original literature. The proportions in each pie chart represents proportion of pathogen taxa from each collection site. Map lines delineate study areas and do not necessarily depict accepted national boundaries. The data and code needed to generate this figure can be found in https://doi.org/10.6084/m9.figshare.22060646.v7. Base layer of the map is from the Natural Earth 1:50 m map (https://www.naturalearthdata.com/), under a non-exclusive license. (PDF) [file pbio.3002900.s004.pdf]

## Model-averaged importance of terms

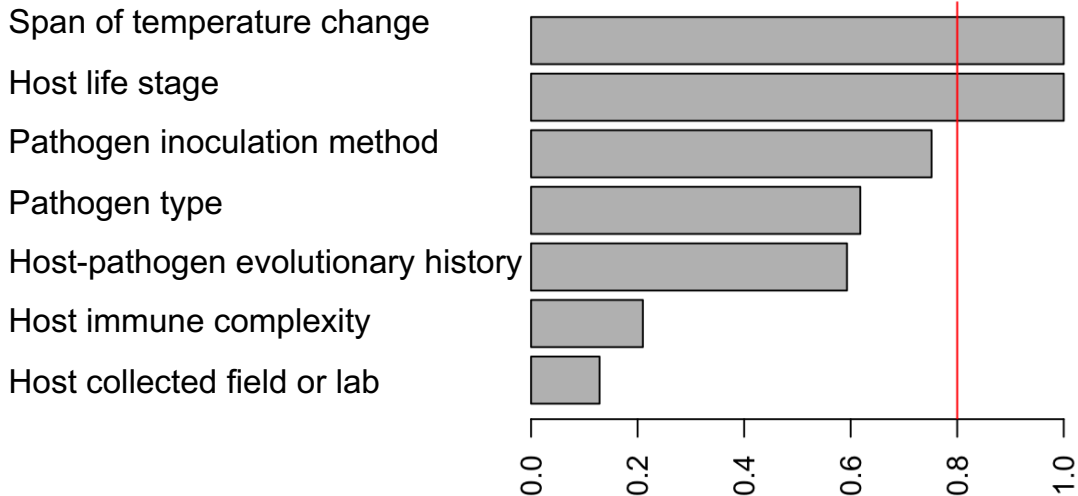

Supplement: S4 Fig — Moderators including “host immune complexity” and “host collected field or lab” with low importance values (model-averaged importance <0.25 and 95% CI included zero) were considered to have little support for inclusion in the model. The data and code needed to generate this figure can be found in https://doi.org/10.6084/m9.figshare.22060646.v7. (PDF) [file pbio.3002900.s005.pdf]

A

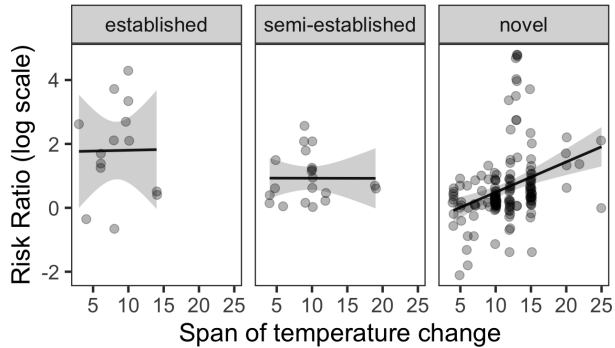

B

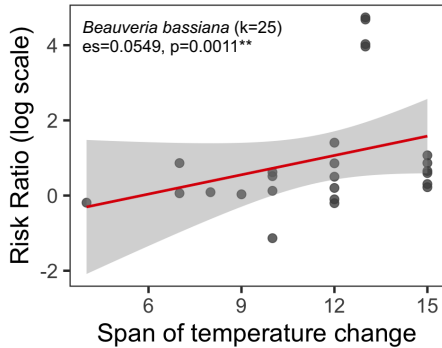

Supplement: S5 Fig — (A) Temperature change is interacting with host–pathogen evolutionary history to influence effect sizes. (B) Increases in effect sizes are associated with larger temperature changes across studies of the pathogen Beauveria bassiana (k: 25 effect sizes across multiple hosts). The data and code needed to generate this figure can be found in https://doi.org/10.6084/m9.figshare.22060646.v7. (PDF) [file pbio.3002900.s006.pdf]

A

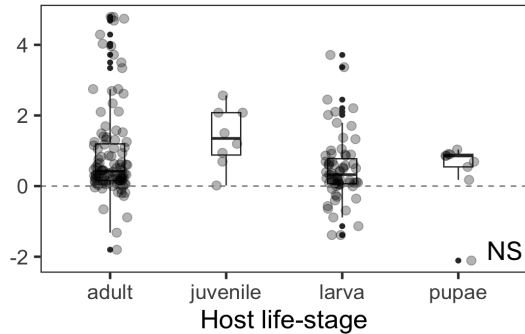

B

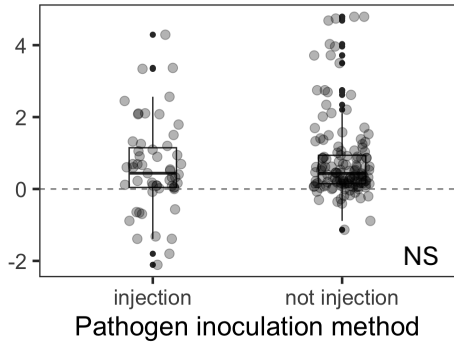

Supplement: S6 Fig — Effect sizes were not impacted by (A) host life-stage, or (B) pathogen was inoculated by injection or other means. Individual effect sizes are displayed as jittered points. The data and code needed to generate this figure can be found in https://doi.org/10.6084/m9.figshare.22060646.v7. (PDF) [file pbio.3002900.s007.pdf]

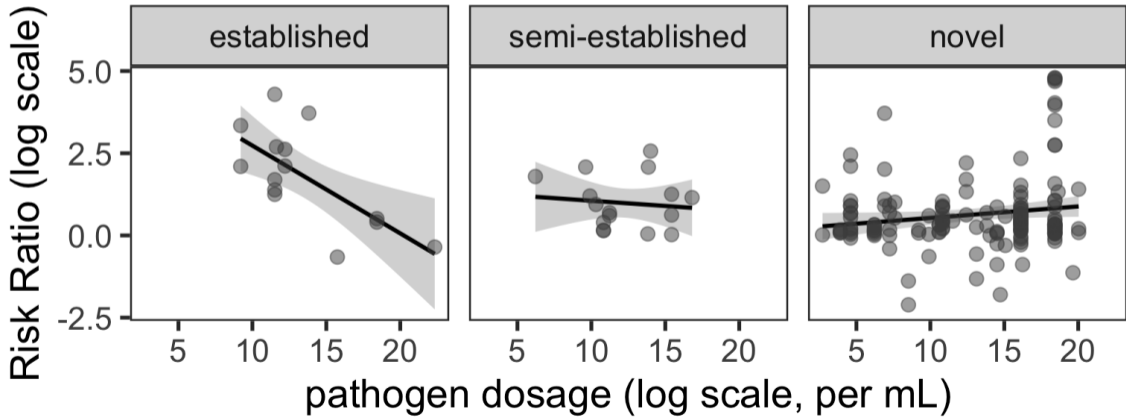

Supplement: S7 Fig — Positive interaction effect between pathogen dosage and semi-established system is observed. The data and code needed to generate this figure can be found in https://doi.org/10.6084/m9.figshare.22060646.v7. (PDF) [file pbio.3002900.s008.pdf]
