# Supplementary material for: Excess mortality of infected ectotherms induced by warming depends on pathogen kingdom and evolutionary history
Source: PLoS Biol. 2024 Nov 18;22(11):e3002900. doi: 10.1371/journal.pbio.3002900 (PMC11611255; doi:10.1371/journal.pbio.3002900)
Supplement: Attachment — Submitted filename: Response_to_reviewers_PlosBio_v3.docx [file pbio.3002900.s013.docx]

REVIEWERS' COMMENTS:

Reviewer #1:

This meta-analysis tackles an overlooked potential complication of climate change: worsening disease severity in animals. It is based on a data set across ectotherms. It nicely takes a very conservative approach, focusing only on host death. This means that the effect of raising temperature is likely even greater if measures of morbidity had been included. The introduction frames the broader question and provides a quick review of possible mechanisms for why increased temperature can worsen disease.

The search terms and inclusion criteria were thoughtfully chosen and defended. I especially liked that they excluded unnatural temperature extremes that would likely bias the outcomes. The handling of death in controls and choice of data collection point and the inclusion of an evolutionary history designation were both great. Moderator variables were also thoughtful.

We thank Reviewer 1 for these positive comments!

Minor corrections/suggestions

Line 86. Novel transmission opportunities? Or do you mean host jumping?

Yes, we meant host jumping, we have now clarified it (line 83).

Line 88. Alter or reduce? You mean reduce, right if the second half of the sentence is true. Then be specific.

Thank you. We have now revised it (line 85).

Differentiate the following two statements better. Confusing as written also as some of this is counter to expectation.

Line 287 We found that the effects of excess host death in established, but not novel systems were the most pronounced in host-bacterial pathogen systems

And

293 We also found that host mortality in novel systems was already higher than that in established associations, at low/baseline temperature

Thank you for your suggestion. We have now adjusted our wording to avoid confusion (line 324-329).

In the discussion you should highlight that if morbidity rather than just mortality was included, effects will be even more widespread.

Thank you. We have now highlighted it in Conclusion and future perspective section (line 443-444).

Reviewer #2:

Overall thoughts

This is a very interesting an timely meta-analysis of the role of temperature change on infection-induced mortality. I found the introduction and the manuscript in general to be well-written and a pleasure to read. While the results are interesting, I found the statistical methods in particular to be lacking in detail, and overly reliant on p-values and significance testing. It is difficult to discern the exact models fit and examined from the Main MS, and the Supplementary tables do not clearly outline the different models. Further, the authors do not appear to have adequately adjusted for host or pathogen taxonomy, and as I can see they do not fit any hierarchical effects, which would be appropriate given the multiple levels of replication in their data.

Major points

- Line 221-224: It appears that you have not adjusted for host taxonomy or phylogenetic relationships among taxa other than gross pathogen type, and host Phylum as binary (C

hordata or not). Phylogenetic or taxonomic non-independence is an important aspect to consider in your models. If phylogenetic relationships among your taxa are unavailable, you may include taxonomic non-independence in these hierarchical effects. For example, a taxonomic tree could be easily obtained and converted to a tree with 1 unit distance associated to each taxonomic level (e.g. species to genus, genus to family, family to order, etc...). I strongly suggest you include this as a structuring factor for your species-level hierarchical effects. Further, you may follow the appraoch first introduced by Schmidt et al (2021) (https://onlinelibrary.wiley.com/doi/abs/10.1111/ele.13740), and later shown to be best practice recommended by Cinar et al (2022) (https://besjournals.onlinelibrary.wiley.com/doi/full/10.1111/2041-210X.13760) of including both taxonomically structured and non-taxonomic structured hierarchical effects for both host and pathogen, as using only the Any

attempts to simplify this model, such as using only one of these components may lead to erroneous inferences from the data.

Thank you for your valuable suggestions. We have now incorporated the taxonomic structures of both hosts and pathogens as random effects in the multilevel random-effects model. The taxonomic trees for host and pathogen species were obtained from NCBI Taxonomy common tree. When species-level taxonomy was unavailable, genus-level information was used instead. Additionally, we included non-taxonomic species-level random effects to account for heterogeneity in effect sizes arising from differences between species that are unrelated to evolutionary relatedness, following the approaches suggested in Schmidt et al. 2021 and Cinar et al. 2022. After incorporating these random effects and performing model selection, the significance of the summary effect size was no longer evident, likely because the effect was predominantly driven by bacterial effect sizes (consistent with what we found previously). Our most key findings remain robust: warming exacerbated virulence in bacterial infections. A significant impact of warming was observed in established systems, while novel systems exhibited higher mortality at low/baseline temperatures. And fungal pathogens were more sensitive to temperature shifts relative to their thermal optima. We have revised the Results section accordingly to highlight these results. Methodological details have also been added to the Methods section (line 230-266). The R scripts and data objects used for analyses and figure generation are publicly available on FigShare (<https://doi.org/10.6084/m9.figshare.22060646.v6>) to facilitate reproducibility.

- Line 225-231: It is unclear why you need to do this sub-group analyses rather than fitting a single joint model. For these types of hierarchical models, it is generally considered best practice to fully and jointly model all levels of the data simultaneously. Please provide additional detail on why this was necessary and what was learned compared to fitting a fully hierarchical model. Currently it is unclear from the MS alone what your main model was. Instead, it appears that you conducted multiple independent models.

Thank you for your insightful comments! Initially, we conducted subgroup analyses by fitting multiple models instead of fitting a single joint model because certain moderators had missing data in some studies. For example, pathogen thermal optima were unavailable for some pathogens, and information on exposure time or infection dosage was not provided in some studies. Including all moderators in a single joint model would have led to the exclusion of some effect sizes (ESs) and could have increased the risk of overfitting due to reduced sample size. Sub-group analyses also helped in exploring specific effects within particular groups that might not be apparent in the overall analysis. Such insights might be crucial. We also assessed whether the overall findings are sensitive to the inclusion and exclusion of particular groups.

To address your suggestion while maximizing the number of retained effect sizes, we have now revised our approach as follows. First, we ensured that no multicollinearity was present among our moderators. For the categorical moderator ‘pathogen thermal optima’ and continuous moderators ‘exposure time’ and ‘infection dosage’, which had missing data in some studies, we conducted multiple meta-regressions to examine the heterogeneity in effect sizes explained by these variables. The interactions between moderators were tested based on *a priori* hypotheses only. This approach allowed us to extract valuable information from these analyses despite the missing data. The remaining moderators were included in a full single joint model, accounting for taxonomic and non-taxonomic non-independence for both host and pathogen species by including these factors as random effects. We assessed the model-averaged importance of moderators and performed model selection (models with ΔAICc < 2 as the final model set). Details of this process can be found in the Methods section (line 230-266).

- While I do not support a p-value / "significance" based paradigm of assessing predictors, you do not appear to correct or discuss the issue of multiple model fitting and p-value correction. I suggest you re-do analyses to fit a main joint model, with subsequent sensitivity models, and discuss relative effect sizes and credible / confidence intervals rather than assessing importance based on p-values alone.

Thank you for your valuable comments! We have now redone analyses to fit a main joint model. We included both taxonomic and non-taxonomic species-level random effects for hosts pathogens. Model selection was performed to account for model uncertainty.

In addition to reporting p-values, we evaluated the importance of moderators based on their occurrence in the model selection process and calculated their confidence intervals after averaging the top model set. Details of the revised methodology are provided in the Methods section (line 230-266).

- While you frame temperature changes with respect to pathogen thermal optima, you do not discuss host thermal optima in this context.

We appreciate your suggestion regarding the importance of both host and pathogen thermal optima in predicting infection outcomes under temperature changes. Unfortunately, we were unable to obtain data on host thermal optima from published literature, primarily due to lack of experimental evidence. It is indeed more challenging to measure animal thermal optima under controlled conditions, compared to microbes. This is due to several factors such as that animals have more complex physiology, and measuring their thermal optima often require a broader range of metrics (e.g., survival, fecundity, etc.), making it difficult to standardize across species and experimental conditions. Additionally, animals can exhibit behavioral responses to unfavorable temperatures, which complicates the measurement of their thermal optima, because their performance at different temperatures is influenced by their ability to seek out optimal conditions. We acknowledge the significance of such data in Results and Discussion (line 392-394) and would welcome any future studies that could provide such information to enhance our understanding of this topic.

While you adjust for host mortality due to warming, there is little to no consideration of sub-lethal changes in host responses to shifting temperatures.

We agree that host sub-lethal changes are also important. In this study, we adopted a conservative approach by focusing on host mortality, as it represents the most extreme outcome of host responses and is highly relevant for animal conservation in the context of climate change (line 118-120). We have now highlighted it in Conclusion and future perspective section (line 443-444).

Further, I cannot find mention of the extent to which hosts or pathogens are pre-acclimated at their Topt or to another condition before the experiments took place. Considering this is an important part of the framing of your discussion, it would be good to clarify this in the methods.

Due to the lack of host thermal optima data, information on whether hosts were acclimated to their thermal optima or other environments is limited. In most instances, hosts were either recently collected from the field or reared in the laboratory for multiple generations. To account for the potential acclimation effects, we included a moderator ‘host source’ distinguishing between wild-collected and lab-reared hosts. For pathogens, specific details on acclimation were generally not provided within individual articles, although they were shown to be cultured within their known thermal ranges. We have now clarified this in the methods (line 193-195, line 205-208).

Minor points

- Lines 134-137: For reproducibility and broader context it would be good to state the institution from which the search was performed as this impacts the results of WOS searches (see <https://gehmana.weebly.com/uploads/3/1/9/8/31987469/dallas_gehman_farrell_2018.pdf>).

Thank you. We have added the details of the institution where literature search was performed in Methods section (line 130-131, 134).

Also, I see that the numbers of articles returned by your search, and retained in the meta-analysis are in the SI, but for clarity please also include these numbers in the main manuscript.

Done (line 273-278).

- Lines 148-158: Unclear why all experimental conditions were not included in analyses. The idea that omitting potential relevant data to "maximise the ecological relevant" is not well explained. You could look at the relative difference in temperature between all time points in a study, which would give you some larger statistical power to describe any effects. Any removal of data should be clearly explained and justified, or else this feels like an arbitrary choice.

Thank you for your valuable comments. We have now included a detailed justification for our choices in the Methods section (line 154-159). The decision to exclude certain experimental conditions was based on the aim of to capture the most substantial changes in host fitness by selecting conditions with the largest temperature changes, while avoiding the confounding effects of extreme temperatures and dosages. In studies that included multiple temperature levels, the extreme lowest and highest temperatures often represent conditions that are rarely encountered by hosts and pathogens in natural settings, typically resulting in host death confounding the infection effects. We excluded those extreme treatments to ensure that observed host mortality was attributable to infection rather than extreme temperatures.

- Line 165-166: Please define what "large host mortality" is. Is this above 10%, or some other proportion which was too large to correct for with Abbot's formula?

Yes it means >10% mortality. We have now clarified it (line 169).

- Line 186-187: Were there any cases in which the the study temperature was significantly reduced from T opt? In this case, wouldn't "Away from T opt" not always include temperatures above the T opt? I assume you are always ordering the experimental conditions to reflect a warming of temperature, but in some cases, I expect the experimental conditions may be structured to reflect a progressive cooling of temperature. In either case, some clarification of this would be nice.

Thank you for your suggestions. Yes, we always order the experimental conditions from low to high temperatures, as we are interested in the effect of warming. Therefore, “away from T_opt_” always indicates temperatures above T_opt_. We have clarified it in the manuscript now (line 188-189).

- Line 212-214: Why was a p>0.07 threshold used here?

We apologize for any confusion. The p-value of p>0.07 was not intended as a threshold but rather represents the lowest p-value obtained from all pairwise correlations. We have clarified this to avoid further confusion (line 225).

- Line 249-250: Why do you think your results support a diminishing of host tolerance with increased temperature, rather than the other competing hypotheses you outlined in the introduction?

Since the significance of the summary effect size was no longer evident after incorporating the new random effects and performing model selection, we have removed this sentence.

- Line 286-288: Since this interaction was found to be significant, it would be best to plot this result in the main MS rather than the effects of individual predictors ignoring this effect.

Thank you for the suggestion. We did not emphasize this result due to the limited representation of novel viral infections in our dataset, which included two effect sizes. As such, caution is necessary when generalizing this interaction effect to a broader context. We have now clarified this point in the main text (line 337-338).

- Line 293-299: The finding that higher baseline mortality was found in novel systems is a very interesting (and core) finding! Considering this, I highly suggest you generate a plot showing this difference in baseline mortality for the main MS. Also, some numeric quantification of the difference in baseline rates would be nice to see.

Thank you! We agree that this finding is very interesting, and we have now incorporated it in the main MS with statistics (Fig 2, line 327-328).

- Line 353-356: For fungi, the vast majority of experiments were with "novel" host-pathogen combinations, which you state previously as having larger baseline mortality rates. Could this be causing your lack of effect? In this case, are you justified in assuming that fungi do not result in higher host death under warming conditions?

Thank you for your valuable point! We have confirmed that the lack of overall virulence-enhancing effect by warming for fungal pathogens was not because they caused higher host mortality at baseline temperatures, by directly comparing host mortality at baseline/low temperatures between across pathogen types. And we have highlighted it in the main text (line 332-335).
