# Supplementary material for: Excess mortality of infected ectotherms induced by warming depends on pathogen kingdom and evolutionary history
Source: PLoS Biol. 2024 Nov 18;22(11):e3002900. doi: 10.1371/journal.pbio.3002900 (PMC11611255; doi:10.1371/journal.pbio.3002900)
Supplement: Attachment — Submitted filename: Response_to_reviewers_PlosBio_R2_v2.docx [file pbio.3002900.s014.docx]

Editorial requests:

a) Please change your Title slightly to make it easier to parse: "Excess mortality of infected ectotherms induced by climate change depends on pathogen kingdom and evolutionary history"

As our study specifically investigated the impact of warming during experimentation on pathogen virulence rather than broader climate change, we have revised the title to: “*Excess mortality of infected ectotherms induced by warming depends on pathogen kingdom and evolutionary history*”, to make the title easier to parse. We hope this change resolves the concern.

b) We find the following passage in the Abstract a little hard to follow: "we demonstrate that warming significantly increased the mortality of hosts infected by bacterial pathogens, as well as pathogens established within the host species." Maybe it would be clearer to separate out these two findings?

Done.

c) Please address the remaining concerns raised by reviewer #2.

Done. See Response to Reviewer #2 (page 2-5).

d) Please ensure that you comply with our Data Policy; specifically, we need you to supply the numerical values underlying Figs 1ABC, 2AB, 3B, S2, S3, S4, S5AB, S6AB, S7, either as a supplementary data file or as a permanent DOI’d deposition. I note that you already have an associated Figshare deposition. Please could you clarify whether this contains the data and code needed to recreate the Figures?

The numerical values for all the SI figures can be found in the R Data file provided in the associated Figshare deposition, as well as in Table S2. Additionally, the R script for loading the data file and recreating all figures is included in the deposition.

e) Please cite the location of the data clearly in all relevant main and supplementary Figure legends, e.g. “The data underlying this Figure can be found in S1 Data” or “The data and code needed to generate this Figure can be found in [https://doi.org/10.6084/m9.figshare.22060646.v6"](https://doi.org/10.6084/m9.figshare.22060646.v6%22)
Done.

f) Please make any custom code available, either as a supplementary file or as part of your data deposition.

Done.

REVIEWERS' COMMENTS:

Reviewer #1:

[checked "Accept" with no further comments]

Reviewer #2:

Major comments / concerns

I reviewed a previous submission of this manuscript, and am very happy to see that the authors have address the majority of my methodological concerns, and incorporated my modelling suggestions into the manuscript. However, it is not recommended to exclude hierarchical effects, even if they appear to explain little variation in the model (lines 2412-245). As per Cinar (2022), it is vital to include both taxonomic and non-taxonomic effects in these models to account for non-independence. If they turn out to explain very little variation, then this is fine, but they should still be included in any models. 

While I personally think is not justified to do model selection or model averaging when you have clear hypotheses (which you do!),  if you choose to go forward with your model selection approach (which I also highly recommend against doing), it would be considered statistical best practice to force these four taxa-level hierarchical effects and the study-level hierarchical effect in your "final" model(s).

Thank you for your suggestion. We performed model selection mainly to evaluate the importance of moderators, given their relevance was not fully known, even though we had clear hypotheses. This approach has been validated in other published meta-analyses (e.g., Vicente-Santos et al. 2023). We have now kept the random effects that explain little variation in the models. After rerunning the model, the main results remained unchanged. The SI and R script have been updated accordingly in the Figshare deposition.

Ref: Vicente-Santos, A., Willink, B., Nowak, K., Civitello, D. J., & Gillespie, T. R. (2023). Host–pathogen interactions under pressure: A review and meta-analysis of stress-mediated effects on disease dynamics. Ecology Letters, 26(11), 2003–2020. https://doi.org/10.1111/ele.14319

After addressing this, one major issue methodological remains - the authors have not adequately justified their choice to "maximise the ecological relevance" of the study by excluding low and high temperatures and pathogen inoculation doses per study (Lines 145-161). This was a concern in my previous review, and the authors responded by stating the aim of the study was to "capture the most substantial changes in host fitness by selecting conditions with the largest temperature changes, while avoiding confounding effects of extreme temperatures and dosages". 

The authors' justification for excluding most substantial changes makes sense, but is not justified through any analysis or presentation of data. This justification assumes that extreme values are those not experienced by hosts or pathogens in natural settings, but the authors say in their response letter that do not know the thermal optima for hosts, meaning that you do not know the range of temperatures that would be considered "extreme". In this case I feel they need to show that these extreme temperatures are associated with ill effects on hosts, and this if course would be host specific.

Without data to support it, the authors state in both the review response and the manuscript that in experimental studies the lowest and highest temperatures often represent conditions that are rarely encountered by hosts and pathogens in natural settings, and that these typically result in host death in the absence of pathogen exposure. However, because the authors have stated that they "ensured that all host mortality in the pathogen treatment groups was due to infection rather than other factors", they should be able to include some of the more extreme temperature effects if no host mortality was observed. 

This may seem minor, but is concerning to me that readers may assume the authors have made some arbitrary choices to filter their data to identify a clear effect. The goal of the study is to look at extreme impacts of temperature and pathogen infection, but the authors exclude many of these "extreme" treatments without explicitly defining how extreme these are (e.g. they remove high and low treatments per study without quantifying how extreme these are, or how extreme they are with respect to "natural" conditions"). If this is not possible, a "sensitivity analysis" including all treatments could be included in the supplement as a comparison.

Thank you for your comment. We agree that excluding the temperature-induced host mortality would also result in the exclusion of most extreme temperatures. After a thorough review of each excluded treatment, we confirmed that all exclusions are justified based on the following criteria:

a) Host mortality was attributable to temperature rather than infection, as seen with the highest temperature treatments in Bally and Garrabou, 2007, Hu et al., 1996, Garcia-Solache et al., 2013, and Shapiro-Ilan et al., 2014; and both the highest and lowest temperatures in Kirk et al., 2018 and Aatif et al., 2020.
b) No pathogen growth or infectivity at extreme temperatures, such as the lowest temperature in Bally and Garrabou, 2007 and Shapiro-Ilan et al., 2014; and both the lowest and highest temperatures in Grewal et al., 1996, Del Valle et al., 2014, and Wekesa et al., 2010.
c) The original study explicitly showed that the extreme temperatures were outside the realistic range for the host-pathogen system, such as in Heinig et al., 2015.

We have now clarified this point in Methods (line 161-165).

Minor comments / concerns

- Line 89: I'm not sure what "Higher virulent temperatures" means - do you simply mean "higher temperatures"?

Yes, revised (line 90).

- Line 99: Grammatical error: "changes" should be "change"

Done (line 100).

- Lines 131-133: You describe the search terms as "keywords", which is confusing. It is unclear which categories of the WOS Core Collection you are searching with these terms. Did you restrict to searching paper keywords with your search terms, or did you use abstracts, or titles, or the proprietary "Topic" field in WOS? This is important for reproducibility, and clarity of your methods. Further, you say you uses two search strings, which were "paired". By "paired", I assume you are linking these terms via an AND statement (e.g. "'parasit*' OR 'pathogen*' AND 'temperature' AND 'virulen*'). Please clarify the exact search string you used.

We have now clarified the search query and the chosen WOS fields (line 132-134).

- Line 237-238: Thank you for incorporating both taxonomic and non-taxonomic effects. As this is a somewhat new best practice for comparative models, please cite both Schmidt et al (2021) (https://onlinelibrary.wiley.com/doi/abs/10.1111/ele.13740), and Cinar et al (2022) (https://besjournals.onlinelibrary.wiley.com/doi/full/10.1111/2041-210X.13760) to give proper credit.:

Something like "as first proposed in Schmidt et al. (2018) and later shown to be best practice by Cinar et al. (2022)", would be fantastic - thank you.

Done (line 244-245).
